# Regular Exercise Improved Fatigue and Musculoskeletal Pain in Young Adult Psoriatic Patients without Psoriatic Arthritis

**DOI:** 10.3390/nu15214563

**Published:** 2023-10-27

**Authors:** Antonio J. Diaz, Miguel A. Rosety, Jose C. Armario, Manuel J. Bandez, Natalia Garcia-Gomez, Eduardo Sanchez-Sanchez, Jara Diaz, Cristina Castejon-Riber, Marco Bernardi, Manuel Rosety-Rodriguez M, Francisco J. Ordonez, Ignacio Rosety

**Affiliations:** 1 School of Nursing and Physiotherapy, University of Cadiz, Ana Viya Avenue, 52, 11009 Cadiz, Spain; antoniojesus.diaz@uca.es; 2 School of Sports Sciences, University of Cadiz, Rep. Saharahui Avenue, 12, 11519 Cadiz, Spain; luna_nueva17@hotmail.com; 3 Dermatology Unit, School of Medicine, University of Cadiz, Fragela Square s/n, 11003 Cadiz, Spain; josecarlos.armario@uca.es; 4 Biomedicine Department, School of Medicine, University of Cadiz, Fragela Square s/n, 11003 Cadiz, Spain; manuel.bandez@uca.es; 5 Histology and Pathology Department, School of Medicine, University of Cadiz, Fragela Square s/n, 11003 Cadiz, Spain; natalia.garcia@uca.es; 6 School of Nursing, Punta de Europa Hospital, Algeciras, 11207 Cádiz, Spain; eduardo.sanchez.sanchez.sspa@juntadeandalucia.es; 7 School of Education Sciences, University of Cordoba, C/San Alberto Magno s/n, 14071 Cordoba, Spain; ccastejon@uco.es; 8 School of Sports Medicine, University La Sapienza, Ple. Aldo Moro 5, 00185 Rome, Italy; marco.bernardi@uniroma1.it; 9 School of Sports Medicine, University of Cadiz, Virgen del Carmen s/n, 11100 Cadiz, Spain; manuel.rosety@uca.es (M.R.-R.M.); franciscojavier.ordonez@uca.es (F.J.O.); 10 Human Anatomy Department, School of Medicine, University of Cadiz, Fragela Square s/n, 11003 Cadiz, Spain; ignacio.rosety@gm.uca.es

**Keywords:** physical activity, musculoskeletal health, nutrition, psoriasis, fatigue

## Abstract

Fatigue and musculoskeletal pain are also frequent in patients with psoriasis (PsO) without arthritis (PsA). The current study aimed to assess the impact of an intervention program based on aerobic training to reduce fatigue and musculoskeletal pain in patients with PsO without PsA. A total of 118 male patients with PsO volunteered in the current interventional study and were randomly allocated to the experimental (*n* = 59) or control group (*n* = 59). The intervention consisted of a 16-week aerobic training program on a treadmill, three sessions per week, consisting of a warm-up, 35–50 min treadmill exercise (increasing 5 min/4 weeks) at a work intensity of 50–65% of peak heart-rate (increasing 5%/4 weeks), and cooling-down. The functional assessment of chronic illness therapy fatigue scale (FACIT-Fatigue), health assessment questionnaire disability index (HAQ-DI), and visual analog scale (VAS) were compared pre and post intervention. Nutritional intake, maximal aerobic power, lipid profile, serum markers of muscle damage, and body composition were also assessed. When compared to baseline, FACIT-Fatigue, HAQ-DI, and VAS scores were significantly improved without increasing markers of muscle damage. Fat mass percentage, lipid profile, and maximal oxygen consumption were also improved. In conclusion, a 16-week aerobic training program at moderate intensity was safe, well tolerated, and effective in psoriatic patients without PsA. Long-term follow-up studies are required to examine whether these promising results may improve clinical outcomes.

## 1. Introduction

Psoriasis (PsO) is a chronic, immune-mediated inflammatory skin disease with a high prevalence in Western societies [1]. Recent studies have emphasized that psoriasis is a systemic disease with multiple comorbidities that extend beyond the skin [2,3,4]. It has been associated with an increased risk for comorbidities, including but not limited to arthritic, psychological, and cardiometabolic conditions [2,3,4,5]. In a more detailed way, the overall pooled prevalence of metabolic syndrome in adult patients with PsO was 32% (95% confidence interval (CI), 0.26–0.38) [6]. These findings could be explained at least in part because patients with PsO revealed excessive ectopic fat accumulation when compared to BMI-matched controls without PsO [7]. As a consequence, visceral fat should be considered a key target not only for pharmacological treatments but also for lifestyle interventions in this population group [7].

However, musculoskeletal symptoms, including pain and fatigue, are highly prevalent in patients with PsO, having a negative impact on their physical function and quality of life [8]. These symptoms have been described not only in adults but also in children and adolescents with PsO [9]. In a more detailed way, Merola et al. [8] reported an increased prevalence of musculoskeletal symptoms in a 60-month follow-up study of newly diagnosed adult patients with PsO (47.1% at baseline, 48.2% after 12 months, and 82.1% after 60 months). The same authors also found that baseline musculoskeletal symptoms were correlated with an increased risk of a PsA diagnosis [8]. Similarly, a recent prospective cohort study demonstrated the existence of nonspecific musculoskeletal symptoms preceding the onset of psoriatic arthritis (PsA) amongst patients with PsO [10]. In fact, up to 30% of patients with PsO may develop psoriatic arthritis (PsA) during their lifetime [11]. This finding is of particular interest because PsA may interfere with daily routines, leisure/sports activities, and work/education in a serious manner [12,13]. In summary, musculoskeletal pain is frequently observed in patients with psoriasis both with and without PsA [14,15]. In this respect, it should be highlighted that musculoskeletal pain may affect the quality of life of patients with PsO to a level similar to that of patients with PsA [2]. Surprisingly, this issue has received little attention in both the medical literature and clinical settings.

For the reasons already mentioned, PsO may cause great physical, emotional, and social burdens that inevitably lead to considerable use of medical care and economic resources in both private and public health services [16,17]. Accordingly, a comprehensive and multidisciplinary approach involving not only dermatologists but also general practitioners is required to achieve better health outcomes for these patients in a timely manner. [16,18]. In this regard, evidence-based guidelines for the treatment of PsA recommend exercise as a non-pharmacological therapy to improve functional capacity [19,20]. 

In a more detailed way, increasing physical activity has been inversely associated with the risk of psoriasis, as the most physically active quintile of participants during a 1,195,703 person-years of follow-up (14 years, 1991–2005) had a lower multivariate relative risk (RR) of PsO (0.72 (95% CI, 0.59–0.89; *p* < 0.001)) [21].

Additionally, high levels of physical activity have been shown to reduce the risk of PsA in patients with PsO in a recent population-based study in Norway [22]. On the other hand, recent meta-analyses have suggested that higher BMI increased the odds of psoriasis occurrence [23]. Furthermore, obesity has been also associated with increased severity of PsO and lower response to modern biological treatment [23]. 

In spite of the above-mentioned pieces of evidence, several studies have demonstrated that patients with PsO generally exhibited more sedentary behavior and were less physically active than matched healthy controls [24]. In a more detailed way, the study of 2011–2014 NHANES (national health and nutrition examination) data of 9174 patients demonstrated that those with PsO were significantly less likely to engage in moderate to vigorous physical activity in comparison with patients who did not suffer from PsO (16.1% vs. 28%; *p* = 0.042). Similarly, other survey-based studies focused on the international physical activity questionnaire (IPAQ) also found that patients with PsO were significantly less physically active than healthy controls [25]. In any case, this fact could be of special clinical interest provided that sedentary lifestyle may influence not only the psoriasis natural course but also the existence of comorbidities [24].

Finally, this study aimed to assess the influence of an intervention program based on aerobic training on reducing fatigue and musculoskeletal pain in adult patients with psoriasis without PsA. The secondary objective was to determine the effects of the intervention on body composition, maximal aerobic power, and serum muscle damage markers.

## 2. Materials and Methods

### 2.1. Study Design and Participants

A total of 118 patients with psoriasis volunteered for the current interventional study which used a pre-/post-test design. The adequacy of sample size was tested using the statistical software Granmo v7.12 (IMIM, Barcelona, Spain) with an accepted two-sided alpha risk of 0.05 and a beta risk of 0.2. In addition, a loss-to-follow-up rate of 10% was estimated. 

All participants met the following inclusion criteria: male; 18–45 years of age; a diagnosis of mild psoriasis on the basis of the PASI (psoriasis area severity index) and DLQI (dermatology life quality index) scores < 7 [26]; topical psoriasis treatment (excluding steroids at least in the last 3 months); and medical approval for participation in physical activity, conducted by an experienced sports medicine physician. The exclusion criteria were as follows: systemic antipsoriatic therapy; patients with plantar psoriasis; comorbidities that might have an impact on their ability to participate in an exercise program such as PsA; regular intake of statins; regular consumption of antioxidant supplements; history of smoking or alcohol intake; participation in a training program in the six months prior to participation in the trial; and not completing at least 90% of the training sessions in the trial.

After reviewing the inclusion/exclusion criteria and signing the informed consent form, the participants were randomly allocated (in a 1:1 ratio) to the experimental (*n* = 59) or control (*n* = 59) group using a computer-generated randomization scheme.

### 2.2. Intervention

Participants in the intervention group participated in a 16-week aerobic training program on a conventional motorized treadmill, for three sessions per week, consisting of a warm-up (10–15 min), 35–50 min treadmill exercise (increasing 5 min each four weeks) at a work intensity of 50–65% of peak heart rate (increasing by 5% every four weeks) measured during a previous maximal treadmill test, and cooling-down (5–10 min). In order to ensure that the training workload was appropriate during the entire session, all participants from the intervention group wore a wireless wearable heart rate monitor (Sport Tester PE3000, Polar Electro, Kempele, Finland).

### 2.3. Outcomes

#### 2.3.1. Patient-Reported Outcomes

##### Functional Assessment of Chronic Illness Therapy Fatigue Scale (FACIT-Fatigue)

The FACIT-Fatigue scale is a valid and reliable instrument for measuring fatigue, which has also been validated in patients with psoriasis [27]. Responses to the 13 items of the FACIT-fatigue questionnaire were each measured on a 4-point Likert scale. Thus, the total score ranged from 0 to 52. A high score indicated less fatigue. 

##### Health Assessment Questionnaire-Disability Index (HAQ-DI)

This index consists of 20 items, ordered into eight domains to investigate the limitations in performing daily physical activities. The highest item score within each category is used as the score for that category. Finally, the scores for the categories are averaged to construct a single total score ranging from 0 (no disability) to 3 (completely disabled). This scale has been widely used in patients with PsA, both in the literature and in clinical settings [28]. 

##### Visual Analog Scale (VAS)

Pain intensity was measured using a visual analog scale (VAS). Patients were asked to rate the pain they experienced during the last week on a 100 mm line anchored by two descriptors: 0 meaning “no pain” and 100 meaning “unbearable pain”. A change of >10 mm in the VAS score mm has been considered a clinically important difference in previous studies that focused on patients with psoriatic arthritis [29].

#### 2.3.2. Biochemical Outcomes

Blood samples were collected from the antecubital vein into tubes containing EDTA after a 12 h fast. The samples were centrifuged at 3000 rpm for 20 min in a clinical centrifuge. Plasma was separated and stored at −80 °C until further analysis. 

The levels of plasma glucose and lipid profile (high-density lipoprotein-cholesterol (HDL-cholesterol); low-density lipoprotein-cholesterol (LDL-cholesterol); triglycerides) were measured by spectrophotometry (Advia 2400, Siemens HealthCare Diagnostics, Deerfield, IL,USA). Serum samples were also analyzed using one-step sandwich immunoassays for creatine kinase activity and myoglobin concentration (AU5800 Plus Biochemical Autoanalyzer, Beckman Coulter Inc., Brea, CA, USA) as markers of muscle damage. These outcomes were assessed in each participant at baseline (week 0) and 24 h after the final training session scheduled in the last week (week 16) of the program.

#### 2.3.3. Aerobic Exercise Power

All participants underwent a modified Bruce multistage maximal treadmill protocol [30], first at baseline (week 0) and, finally, one week after the end of the study. Briefly, the protocol consisted of two warm-up stages, each lasting 3 min. The first was at 1.7 mph (2.74 km/h) and 0% gradient, and the second was at 1.7 mph and 5% gradient. The slope of the treadmill was increased by 2% at three minute intervals, and the speed was also increased in accordance with the 10-stage protocol (2.5, 3.4, 4.2, 5.0, 5.5, 6.0, 6.5, 7.0, and 7.5 mph (4.02, 5,47, 6.76, 8.05, 8.85, 9.65, 10.46, 11.26, and 12.07 km/h)). The test was discontinued when the participant could not continue running due to physical exhaustion. Blood pressure and cardiac electrical activity were continuously monitored for all patients. Maximal oxygen consumption (VO_2_max) was measured and calculated directly using a gas analyzer (Cosmed Fitmate Med, Rome, Italy). Before each test, the gas analyzers and turbine flowmeter of the system were calibrated according to the manufacturer’s instructions.

#### 2.3.4. Body Composition 

Body composition was assessed using a bioelectrical impedance analysis (BIA; Tanita TBF521) after an overnight fast. The participants were requested not to perform any moderate or vigorous exercise for 24 h before testing and to abstain from eating or drinking for 2 h before testing. They were also asked to urinate immediately before the collection of the samples. Measurements were obtained while the participant was standing erect and barefoot on the analyzer’s footpads and wearing either a swimsuit or undergarments. Total body water (TBW) was estimated using the equation provided by the manufacturer. The instrument was calibrated before each evaluation using known resistors. Fat mass was determined using the equation devised by Sun et al. [31], which was designed for a large population and a broad range of BMI values. In this respect, free fat mass = −9.529 + 0.696 × (height^2^/resistance) + (0.168 × weight) + (0.016 × resistance). Fat mass was calculated as the difference between body weight and free fat mass. The intraclass correlation coefficient for fat mass was 0.98 (0.97–0.99).

#### 2.3.5. Nutritional Intake Record

To control for the potential confounding effect of diet, participants were carefully instructed to complete a food consumption frequency questionnaire for three days (two weekdays and one weekend day). Energy and nutrient intakes were calculated using specific software (VD-FEN 2.1, Madrid, Spain) based on updated Spanish food composition tables [32].

No significant difference was found between the intervention and control groups when assessing energy intake (2361 ± 241 vs. 2196 ± 212 kcal; *p* = 0.31). Furthermore, the mean daily vitamin intake showed no significant differences (10.8 ± 2.6 vs. 10.2 ± 2.3 mg/d vitamin E; *p* = 0.16; 87.6 ± 24.4 vs. 82.1 ± 22.8 mg/d vitamin C; *p* = 0.36). 

### 2.4. Ethics and Statistics

The study protocol complied with the principles of the Declaration of Helsinki (2013). Written informed consent was obtained from all participants. The current protocol was approved by the Institutional Ethics Committee of Cadiz (Cadiz, Spain). The results are expressed as mean (SD). The Shapiro–Wilk test was used to assess whether the data were normally distributed. To compare the mean values, one-way analysis of variance (ANOVA) with post hoc Bonferroni correction was used to account for multiple tests. For all tests, statistical significance was set at an alpha level of 0.05. 

## 3. Results

Firstly, the baseline characteristics of psoriatic patients in the intervention and control groups are listed in Table 1.

No dropouts or sport-related injuries were reported during the study period. Additionally, the overall mean adherence rate was excellent (92%).

When compared to baseline, the experimental group significantly improved their maximal aerobic power (VO_2_max) (22.5 ± 5.5 vs. 25.4 ± 4.8 mlO_2_/kg/min; *p* = 0.020). On the contrary, no changes were found in the control group (21.4 ±5.1 kg vs. 21.0 ± 5.3 mlO_2_/kg/min; *p* = 0.286). Additionally, the intervention also improved fat mass percentage (35.9 ± 4.4% vs. 33.8 ± 4.1%; *p* = 0.016) and BMI (31.7 ± 3.9 vs. 30.3 ± 3.7 kg/m^2^; *p* = 0.039) in the experimental group. As was expected, no significant changes were reported in the control group for both items (36.6 ± 4.8% vs. 37.0 ± 4.2%; *p* = 0.156, and 32.2 ± 4.1 vs. 32.7 ± 4.0 kg/m^2^; *p* = 0.402, respectively).

The serum lipid profile was significantly improved after the completion of the intervention program (47.0 ± 7.1 vs. 54.3 ± 6.8 mg/dl HDL-cholesterol; *p* = 0.006; 146.4 ± 17.4 vs. 129.6 ± 16.1 mg/dl triglycerides; *p* = 0.021). Conversely, no significant changes were found when assessing glycemia (109.8 ± 8.6 vs. 103.4 ± 7.9 mg/dl; *p* = 0.208). In the control group, neither serum lipid profile nor glycemia were changed in a significant manner. These results are summarized in Table 2. Lastly, the aerobic training caused no significant changes in serum markers of muscle damage after the completion of the intervention program (307.7 ± 41.8 vs. 326.0 ± 39.5 U/L CK; *p* = 0.262; 45.2 ± 6.3 vs. 48.0 ± 5.9 ng/mL Mb; *p* = 0.316). None of these changes were statistically significant in the control group, and they are listed in Table 2.

With regard to the patient-reported outcomes, the mean FACIT-fatigue score was significantly increased after completing the intervention program (39.2 ± 9.4 vs. 44.5 ± 8.6; *p* = 0.027). Similarly, the experimental group also improved the HAQ-DI score (0.59 ± 0.18 vs. 0.47 ± 0.13; *p* = 0.034). Lastly, VAS score was significantly decreased at the end of the intervention (27.8 ± 15.2 vs. 20.6 ± 14.8; *p* = 0.009). Conversely, no significant changes in any of the above-mentioned outcomes (FACIT; HAQ-DI; VAS) were reported in the control group (Table 3).

## 4. Discussion

Increasing evidence suggests that PsO is a high-impact chronic disease and, therefore, it is fundamental to advocate a multidisciplinary approach to obtain better health outcomes [16]. The present study was the first to evaluate the influence of aerobic training on the musculoskeletal system in patients with psoriasis who do not have PsA. As hypothesized, the intervention program significantly improved both fatigue and musculoskeletal pain. Similarly, in a recent study, Thomsen et al. [29] reported that patients with PsA tolerated high-intensity interval training (HIIT) without deterioration of disease activity and with a significant improvement in fatigue. This finding was of particular interest given that the improvement of fatigue has been considered a priority by patients with PsO [33] and with PsA [34]. These promising results were also reported in a systematic review that emphasized that physical activity programs offered clear benefits on fatigue for people with chronic inflammatory conditions, such as arthritis [35].

In addition, it should be emphasized that aerobic training did not significantly increase serum markers of muscle damage in the experimental group. Additionally, no dropouts or sport-related injuries were reported during the study period. These results suggest that aerobic training performed at moderate intensity is safe for this population; therefore, neither fatigue nor pain should be considered a barrier to physical activity in patients with psoriasis without PsA [36,37]. In contrast, serum markers of muscle damage have been reported to have increased in patients with PsA treated with a first-line, biologic therapy etanercept [38] and the Janus kinase (JAK) inhibitor upadacitinib [39].

Improvement in both fatigue and pain may ultimately lead to improved function in patients with psoriasis [19,40,41]. Similarly, a recent systematic review and meta-analysis concluded that exercise interventions could reduce musculoskeletal pain which may lead to improved function in patients with other rheumatic and musculoskeletal diseases (RMDs) [42]. Consequently, these recommendations should guide shared decision making between patients with RMDs and healthcare providers when developing and monitoring multidisciplinary treatment plans [43].

In this respect, the present study also demonstrated that the intervention improved maximal oxygen consumption in the experimental group. The latter finding could also be explained, at least in part, by the decreased fat mass percentage observed on completion of the program. In a more detailed way, our results also demonstrated that a 16-week aerobic training program improved lipid profile in adult patients with PsO. In clinical practice, similar results have been reported when using classical (statins) and new (glucagon-like peptide-1 (GLP-1) receptor agonists; pioglitazone) hypolipidemic drugs on psoriasis [44]. These results could be of great interest given the link between cholesterol metabolism and psoriatic chronic inflammation reported in recent studies [45]. However, it should be highlighted that statin therapy may increase muscle pain and weakness [46] whereas the current intervention based on aerobic training may improve both pain and fatigue in patients with PsO.

In a previous study, Thomsen et al. [47] reported that a 3-month intervention program based on HIIT significantly improved not only oxygen consumption (expressed as VO_2_max) but also truncal fat percentage in adult patients with PsA. It should be highlighted that the follow-up of these patients showed that these promising results were maintained for at least up to 9 months after completing the intervention program [47]. In a later study, the same research group found no evidence of inflammation in peripheral joints and entheses in patients with PsA after completing structured HIIT for 11 weeks [48]. And even more pieces of evidence are available when reviewing the literature suggesting that HIIT is safe for patients with PsA [49].

In this respect, the reduction in fat mass may be of great interest to these patients, as visceral fat has been shown to be strongly associated with disease activity in psoriasis [7]. Similarly, weight loss induced by an intervention based on a very low-energy diet (daily intake of 640 kcal) was associated with sustained improvement in disease activity after a 24-month follow-up period [50]. Regarding potential confounding effects of diet in the present study, no significant differences regarding daily total energy or antioxidant vitamin intake were found between the intervention and control groups. Surprisingly, a previous study found that adult patients with PsO had a poor nutritional status, and were at risk of nutrient deficiencies compared to BMI-matched controls without PsO [5]. On reviewing the literature on this population group, it appears that future studies focusing on interventions based on the combination of a healthy anti-inflammatory personalized diet and exercise are urgently required [51].

The high prevalence of being overweight and obese among patients with psoriasis may contribute to the increased cardiovascular disease risk reported in previous studies [23,52]. Visceral adiposity may be a more pressing concern in PsO relative to PsA, because visceral fat accumulates more in patients with PsO alone than in patients with PsA [52]. Jarret [53] concluded that dermatologists and general practitioners should play an additional role in informing patients with PsO about their increased cardiovascular risk. For the reasons already mentioned, Blake et al. [7] pointed out that fat mass could be an important future target for both pharmacological and lifestyle interventions. In fact, our study demonstrated that regular exercise could be an effective and safe lifestyle intervention to achieve this goal. 

In this respect, the location of skin lesions should be taken into account when designing specific interventions based on physical activity for this population group. In a more detailed way, palmoplantar psoriasis (PPP) is a disabling and challenging type of *PsO* that affects the skin of the palms and soles typically in a bilateral and symmetrical way. Although the prevalence of PPP in patients with PsO may be as low as 2.8% [54], these patients suffer from significantly higher impairment during both work and leisure time [55]. In the current design, focused on treadmill running, patients with plantar PsO were excluded and this fact may have contributed, at least partially, to the excellent adherence rate observed during the intervention.

Finally, the current study has some limitations. A major limitation was the relatively short duration of the exercise intervention and the lack of follow-up to determine the optimal duration of the aerobic training for the positive effects to be maintained. Additionally, it would have been of interest to assess the effect of the intervention not only on fat mass percentage but also on fat mass distribution given the key role of visceral fat mass on psoriatic comorbidities. Lastly, the fact that women did not participate in the present study may mean that the current results have a weaker applicability in clinical practice. The current intervention focused just on male patients was based on the fact that pain and fatigue are more severe and frequent amongst female patients with PsO, so more withdrawals and injuries could be expected [56]. In addition, previous studies have also reported a higher incidence and prevalence of PsA in male patients [57]. Consequently, future studies ensuring the appropriate enrollment of women are still required to confirm the current promising results in male patients with PsO.

On the other hand, the strengths of the current study include the excellent adherence rate and safety of aerobic training for adults with PsO. In addition, a larger (*n* = 118 vs. *n* = 67) and more homogeneous (118 men vs. 43 women plus 27 men) study population was enrolled than that in a previous study on this topic [29]. It should also be noted that the threshold for disease activity (PASI-1) was among the inclusion criteria for the study. This intervention can easily be reproduced using a conventional motorized treadmill at home. In fact, in a recent study that focused on barriers to physical activity, it was found that patients with PsO may fear the negative reaction of other people to their visible skin lesions and consequently, they were less likely to exercise in fitness centers and swimming pools [36]. Similarly, children and adolescents with this condition have been reported to be stigmatized by peers, so their recreational activity could be also severely affected [58]. Lastly, given that psoriatic skin lesions also interfere with sweating [59], the control of temperature and humidity during exposure to exercise may play a key role in guaranteeing participants’ comfort and reducing the risk of heat intolerance. Accordingly, treadmill running in climate-controlled environments was an adequate intervention design that may explain, at least in part, the excellent adherence rate in the current study.

Finally, we concluded that a 16-week moderate-intensity aerobic training program reduced fatigue and muscle pain in patients with psoriasis without PsA. In addition, it improved aerobic power and body composition. Further, more long-term follow-up studies are required to determine whether the effects induced by aerobic training may improve clinical outcomes in patients with psoriasis without PsA.

## Figures and Tables

**Table 1:** Baseline characteristics of psoriatic patients in the intervention and control groups.

Characteristics	Intervention Group (*n* = 59)	Control Group (*n* = 59)
Age (years)	35.0 (6.1)	37.8 (6.6)
PASI	4.18 (2.02)	4.40 (2.36)
BMI	31.7 (3.9)	32.2 (4.1)
FM	35.9 (4.4)	36.6 (4.8)
LDL-cholesterol	126.6 (18.3)	131.2 (20.1)
HDL-cholesterol	47.0 (11.4)	45.8 (12.1)
Triglycerides	146.4 (17.4)	151.3 (16.9)
Glycemia	112.8 (8.6)	115.4 (7.4)
VO2max	22.5 (5.5)	21.4 (5.1)
FACIT-fatigue	39.2 (9.4)	37.9 (9.0)
HAQ-DI	0.59 (0.1)	0.61 (0.1)

Note: Results were expressed as mean (SD). PASI: psoriasis area severity index score BMI: body mass index expressed as kg/m^2^; FM: fat mass percentage. LDL-cholesterol, HDL-cholesterol, triglycerides and glycemia were expressed as mg/dl. VO2max: maximal oxygen consumption expressed as mlO_2_/kg/min. FACIT-fatigue: functional assessment of chronic illness therapy fatigue scale; HAQ-DI: health assessment questionnaire-disability index. No significant differences were found in any of the already-mentioned parameters (*p* > 0.05).

**Table 2:** Serum lipid profile, glycemia, and markers of muscle damage at baseline (pre intervention) and at the end of the study (post intervention) in male adults with psoriasis.

Markers	Study Group		Control Group	
	Pre-Test	Post-Test	Baseline	Final
LDL-c	126.6 (18.3)	112.1 (17.7) ^a,b^	131.2 (20.1)	136.4 (21.2)
HDL-c	47.0 (7.1)	54.3 (6.8) ^a,b^	45.8 (7.7)	44.1 (7.9)
TG	146.4 (17.4)	129.6 (16.1) ^a,b^	151.3 (16.9)	154.6 (17.6)
Gly	109.8 (8.6)	103.4 (7.9)	112.4 (7.4)	114.0 (7.5)
CK	307.7 (41.8)	326.0 (39.5)	294.8 (43.2)	301.6 (44.0)
Mb	45.2 (6.3)	48.0 (5.9)	42.9 (7.2)	43.7 (7.5)

Note: Results expressed as mean (SD). LDL-c: low density lipoprotein-cholesterol expressed as mg/dl; HDL-c: high density lipoprotein-cholesterol expressed as mg/dl; TG: triglycerides expressed as mg/dl; Gly: glycemia expressed as mg/dl; CK: creatine kinase activity expressed as U/L; Mb: myoglobin concentration expressed as ng/mL. ^a^ *p* < 0.05 versus pre-test; ^b^ *p* < 0.05 versus the control group (final).

**Table 3:** Influence of the aerobic training program on FACIT-fatigue, HAQ-DI, and VAS scores in psoriatic patients at baseline and after completing the intervention program.

Outcomes	Intervention Group		Control Group	
	Pre-Test	Post-Test	Baseline	Final
FACIT-fatigue	39.2 (9.4)	44.5 (8.6) ^a,b^	37.9 (9.0)	39.4 (8.8)
HAQ-DI	0.59 (0.18)	0.47 (0.13) ^a,b^	0.63 (0.16)	0.66 (0.17)
VAS	27.8 (15.2)	20.6 (14.8) ^a,b^	29.5 (14.7)	29.9 (15.5)

Note: FACIT-fatigue: functional assessment of chronic illness therapy fatigue scale; HAQ-DI: health assessment questionnaire-disability index; VAS: visual analogue scale. ^a^ *p* < 0.05 versus pre-test; ^b^ *p* < 0.05 versus the control group (final).

## Data Availability

The data are collected in a database prepared by the research team.
